# A Retrospective Review of Clinical Characteristics and Risk Factors of Dysphagia in Patients with Dermatomyositis

**DOI:** 10.1007/s00455-024-10763-6

**Published:** 2024-11-08

**Authors:** Ivy Cheng, Christina Sze Man Wong, Henry Hin Lee Chan

**Affiliations:** 1 Division of Dermatology, Department of Medicine, Li Ka Shing Faculty of Medicine, The University of Hong Kong, Hong Kong, China; 2 Division of Dermatology, Department of Medicine, Queen Mary Hospital, Hong Kong, China; 3 Centre for Gastrointestinal Sciences, Division of Diabetes, Endocrinology and Gastroenterology, School of Medical Sciences, The University of Manchester, Manchester, UK; 4 Department of Neurology with Institute for Translational Neurology and Institute for Biomagnetism and Biosignal Analytics, The University of Münster, Münster, Germany; 5 Unit of Human Communication, Learning and Development, Faculty of Education, The University of Hong Kong, Hong Kong, China

**Keywords:** Dermatomyositis, Dysphagia, Myositis, Oesophageal Dysmotility, Risk Factors

## Abstract

Background: Dermatomyositis is a rare autoimmune-mediated disease characterised by distinctive rash and progressive muscle weakness. Patients with dermatomyositis may develop swallowing disorders (dysphagia) due to the inflammation of muscles involved in swallowing which may lead to serious health consequences. However, to date, the clinical characteristics of and risk factors for dysphagia in dermatomyositis remain poorly understood. This retrospective study aimed to identify the characteristics and risk factors for dysphagia in dermatomyositis. Methods: All patients with clinical diagnosis of dermatomyositis (ICD-9-CM 701.3) were identified and retrieved retrospectively via hospital electronic record over a 10-year period for review. Results: A total of 231 patients were identified with 149 fulfilled the inclusion criteria (median age [range] = 54.5 [3–92] years; 51 males) were recruited. The incidence of dysphagia was 18.8%, with predominantly pharyngeal phase impairments. Six patients had silent aspiration. Dysphagia was positively correlated with the age of diagnosis (*r*[148] = 0.187, *p* = 0.023), mortality (*r*[149] = 0.186, *p* = 0.023), presence of underlying malignancy (*r*[149] = 0.222, *p* = 0.007), methylprednisolone use (*r*[149] = 0.166, *p* = 0.042) and intravenous immunoglobulin (IVIg; *r*[149] = 0.217, *p* = 0.008), and negatively correlated with disease duration (*r*[147]=-0.273, *p* < 0.001). Moreover, it was more likely to have symptomatic dysphagia in patients prescribing systemic corticosteroid (OR[95%CI] = 4.43[1.02, 19.27], *p* = 0.047) and IVIg (OR[95%CI] = 6.39[1.14, 35.68], *p* = 0.035). Discussion: Dysphagia was associated with advanced age, increased mortality and malignancy in patients with dermatomyositis. Routine screening of dysphagia is recommended at initial diagnosis and severe disease activity requiring high dose systemic steroid and IVIg use.

## Introduction

Dermatomyositis (DM) is rare autoimmune disease with an incidence of 9.63 per 1 million individuals [1]. It is a form of idiopathic inflammatory myopathy (IIM) and microangiopathy that affects the skin and muscle [2]. In dermatomyositis, activation and deposition of complements may cause lysis of endomysial capillaries and muscle ischaemia [2]. Dermatomyositis is characterised by a distinctive rash such as heliotrope rash, erythematous rash on face, neck and anterior chest (V-sign) or back and shoulders (shawl sign), or raised violaceous rash or papules (Gottron’s papules) over the hand knuckles, accompanied by an inflammation of the musculatures with symmetrical and progressive proximal muscle weakness, and elevated serum levels of muscle-associated enzymes [2–4].

Patients with dermatomyositis may experience difficulties in swallowing (dysphagia). Swallowing is a complex neuromuscular process involving the coordination of over 25 pairs of muscles from mouth to the stomach [5]. In patients with dermatomyositis, the oropharynx and upper third of oesophagus which are consisted of striated skeletal muscle tissues may be affected by inflammation, resulting in dysphagia [6–8]. Dysphagia can lead to serious negative consequences to individuals physical and mental well-being, including malnutrition, dehydration, poor quality of life, aspiration pneumonia, prolonged hospital stay and increased mortality secondary to pneumonia [9, 10]. It can occur as an initial symptom [11, 12], or progressively over the course of dermatomyositis [13, 14].

Early identification of dysphagia symptoms in patients with dermatomyositis allows timely management of dysphagia. This is important for functional recovery of the disease because patients can maintain adequate intake of nutrition and medications necessary for recovery and general well-being. However, to date, dysphagia in dermatomyositis remain poorly understood [6]. Therefore, this study aimed to analyse the characteristics of dysphagia and identify risk factors for dysphagia in dermatomyositis.

## Materials & Methods

This retrospective cohort study was approved by the Institutional Review Board of the University of Hong Kong/ Hospital Authority Hong Kong West Cluster (reference: UW22-628). Clinical data analysis and recording system (CDARS) under Hospital Authority Hong Kong which includes a territory-wide patients’ electronic demographic and medical data were searched for International Classification of Diseases, Ninth Revision, Clinical Modification (ICD-9-CM) code 710.3 to identify adult patients with dermatomyositis and related conditions in outpatient settings in a tertiary hospital, Queen Mary hospital, which serve one-third of population in Hong Kong. The study period was over 10 years from 1 January 2013 to 31 December 2022 in which, patients who were newly diagnosed with dermatomyositis using the diagnostic criteria suggested by Bohan and Peter [3, 4] and Dalakas and Hohlfeld [2], and a histological diagnosis, following up at Queen Mary Hospital Dermatological clinics, were included. Patients who has uncertain diagnosis, absence of histological proof of DM, incomplete medical information such as default follow-up, missing data were excluded.

### Data Extraction

Demographic data which included gender and age, clinical characteristics including the age of diagnosis, disease duration (defined as the duration between date of symptom onset and date of end of follow-up or death), presence of malignancy, interstitial lung disease (ILD), bronchiolitis obliterans organizing pneumonia (BOOP), ischaemic heart disease and stroke, presence of dysphagia and its characteristics and management, laboratory characteristics including level of creatine kinase (CK) before systemic treatment and the duration for CK level to normal level, presence of autoantibodies which included melanoma differentiation-associated protein 5 (MDA5) and transcription intermediary factor 1-gamma (TIF1-γ), levels of anti-nuclear antibody (ANA) and anti-double stranded DNA (anti-dsDNA) antibody, and the use of medications were extracted. Autoantibodies MDA5 and TIF1- γ were chosen as target of interest for this review because these autoantibodies are associated with increased risk of interstitial lung disease [15] and underlying malignancy [16] respectively.

### Statistical Analysis

All statistical analyses were performed using Statistical Package for Social Sciences (SPSS) version 26.0 (SPSS Inc., Chicago, IL, USA). Normality of data was assessed using Shapiro-Wilk test. Subsequent analyses were performed using parametric or non-parametric tests based on the normality of data. Continuous data (expressed as mean ± standard deviation [SD] or median [range]) between groups were analysed using independent *t*-test or Mann Whitney *U* test. Categorical data (expressed as proportions) were analysed using Fisher’s exact test and the results were expressed in odds ratio (OR) (95% confidence interval [CI]). Pearson’s correlation was used to evaluate the relationship between clinical and laboratory characteristics and dysphagia. Based on the results on correlation analyses, factors with a *p* value < 0.1 were entered into multivariate logistic regression model, with OR and 95%CI calculated. All *p* values were two-sided and the significance level was set at *p* < 0.05.

## Results

### Overall Characteristics

**Table 1:** Demographic characteristics of the study cohort (*N* = 149)

Demographics	
Gender (Female: Male)	98 : 51
Age of diagnosis (years)	54.5 (3–92)
**Clinical characteristics**	
Disease duration (months)	96 (3–394)
Number of patients went into ^a^remission	7 (4.7%)
Number of deaths	26 (17.4%)
Underlying malignancy	39 (26.2%)
Interstitial lung disease (ILD)	26 (17.4%)
Bronchiolitis obliterans organizing pneumonia (BOOP)	2 (1.3%)
Ischaemic heart disease	9 (6.0%)
Stroke	8 (5.4%)
**Laboratory characteristics**	
CK level (units/L) before systemic treatment	362 (20–11493)
^*b*^*Elevated CK level*	72 (48.3%)
*Duration for CK level remission (days)*	50.5 (1–4261)
MDA5 positive	17 (11.4%)
TIF1-γ positive	16 (10.7%)
ANA level	1:160 (1:40–1:1440)
^*+*^*Elevated ANA level*	47 (31.5%)
Anti-dsDNA antibody level (IU/ml)	3.6 (0.6–246.4)
^*+*^*Elevated anti-dsDNA level*	7 (4.7%)
**Medications**	
Systemic corticosteroid	
Prednisolone	112 (75.2%)
Methylprednisolone	9 (6.0%)
*Duration of systemic corticosteroid use (days)*	1343.5 (3–8049)
Immunosuppressants	
Azathioprine	41 (27.5%)
Hydroxychloroquine	73 (49.0%)
Methotrexate	14 (9.4%)
Mycophenolate mofetil	9 (6.0%)
Tacrolimus	2 (1.3%)
Tocilizumab	1 (0.7%)
Infliximab	1 (0.7%)
Rituximab	6 (4.0%)
Cyclosporine	2 (1.3%)
Intravenous Immunoglobulin	13 (8.7%)

Values are presented in n (%) or median (range) unless otherwise specified

CK: Creatine Kinase; MDA5: melanoma differentiation-associated protein 5; TIF1-γ: transcription intermediary factor 1-gamma; ANA: anti-nuclear antibody; anti-dsDNA: anti-double stranded DNA

^a^ Remission is defined as free of systemic treatment; ^b^CK > 355U/L for males and > 161 U/L for females; ANA > 1/80; dsDNA level > 30 IU/ml. Reference values were provided by the local laboratory

#### Demographic Characteristics

A total of 231 patients with ICD-9-CM 703.1 were identified; 149 patients (98 females, 51 males) with dermatomyositis who fulfilled the inclusion criteria were included for analysis (Table 1). Eight-two patients without skin or muscle biopsy confirmation or incomplete medical information such as default follow-up or missing data were excluded. The median (range) age of diagnosis was 54.5 (3–92) years.

#### Clinical Characteristics

The median (range) duration of the disease is 96 (3–394) months. Only 7 patients (4.7%) went into clinical remission, which was defined as free of systemic treatment, and all of them did not have dysphagia. The mortality rate was 17.4% (*n* = 26). Thirty-nine (26.2%) patients had underlying malignancies including (in descending order of occurrence): lung (*n* = 12), nasopharyngeal (*n* = 10), breast (*n* = 7), liver (*n* = 5), lymphoma (*n* = 5), colon (*n* = 3), corpus uteri (*n* = 3), urinary bladder (*n* = 2), stomach (*n* = 2), bone (*n* = 2), brain (*n* = 1), fallopian tube (*n* = 1), thyroid gland (*n* = 1), prostate (*n* = 1), ovarian (*n* = 1) carcinoma. Nine of these patients had metastatic malignancy. Furthermore, 26 (17.4%) patients had ILD, which was diagnosed by high resolution computed tomography (HRCT; *n* = 15; 57.7%), computed tomography (CT) of the thorax (*n* = 3; 11.5%) and chest x-ray (*n* = 7; 26.9%). Two (1.3%) patients had BOOP diagnosed using biopsy. Finally, 9 (6.0%) patients had ischaemic disease and 8 (5.4%) has stroke.

#### Laboratory Characteristics

The median (range) creatine kinase level of all patients was 362 (20–11493) units/L. The percentage of patients with elevated CK level before systemic treatment, according to the reference values provided by the local laboratory, was 48.3% (*n* = 72). The median (range) duration for CK level to return to normal was 13 (1–4261) days. Seventeen (11.4%) patients had MDA5 antibody and 16 (10.7%) had TIF1-γ antibody. Forty-seven (31.5%) patients had elevated ANA level, with a median (range) of 1:160 (1:40–1:1440). Seven patients had elevated anti-dsDNA level, with a median (range) of 3.6 (0.6–246.4) IU/ml.

#### Medications

Most patients were treated with systemic corticosteroid, including prednisolone (*n* = 112; 75.2%) and methylprednisolone (*n* = 9; 6.0%). The median (range) duration of systematic corticosteroid use was 1343.5 (3–8049) days. Immunosuppressants, including azathioprine (*n* = 41; 27.5%), hydroxychloroquine (*n* = 73; 49.0%), methotrexate (*n* = 14; 9.4%), mycophenolate mofetil (*n* = 9; 6.0%), tacrolimus (*n* = 2; 1.3%), tocilizumab (*n* = 1; 0.7%), infliximab (*n* = 1; 0.7%), rituximab (*n* = 6; 4.0%) and cyclosporine (*n* = 2; 1.3%) were also used. Furthermore, some patients were treated with intravenous immunoglobulin (IVIg; *n* = 13; 8.7%).

#### Characteristics of Dysphagia

**Table 2:** Characteristics of dysphagia among the study cohort (*N* = 149)

	*n* = 149 (% among the study cohort)
Number of patients assessed by speech therapists presenting with symptomatic dysphagia	40 (26.8%)
Number of patients with dysphagia confirmed by clinical bedside +/-instrumental assessment	28 (18.8%)
**Swallowing assessment methods**	*n* = 28 (% among dysphagic patients)
Clinical bedside assessment	28 (100%)
Instrumental assessment	17 (60.7%)
Videofluoroscopic swallowing study (VFSS)	15 (88.2%)
Flexible endoscopic swallowing study (FEES)	3 (17.6%)
Oesophagogastroduodenoscopy (OGD)	2 (11.8%)
**Dysphagia symptoms as revealed by instrumental assessments**	*n* = 28 (% among dysphagic patients)
Oral phase impairments	9 (32.1%)
Reduced tongue movement	6 (21.4%)
Abnormal chewing pattern	4 (14.3%)
Oral residue	3 (10.7%)
Pharyngeal phase impairments	19 (67.9%)
Reduced tongue base retraction	12 (42.9%)
Delayed swallowing reflex	7 (25.0%)
Reduced laryngeal excursion and epiglottic retroflexion	18 (64.3%)
Reduced pharyngeal wall contraction	10 (35.7%)
Impaired upper oesophageal sphincter relaxation	10 (35.7%)
Pharyngeal residue	14 (50.0%)
Penetration	10 (35.7%)
Aspiration	11 (39.3%)
Silent aspiration	6 (21.4%)
Oesophageal phase impairments	5 (17.9%)
Oesophageal dysmotility	4 (14.3%)
Oesophageal residue	2 (7.1%)
**Dysphagia management**	
Enteral feeding	10 (35.7%)
Percutaneous endoscopic gastrostomy (PEG) tube	1 (3.6%)
Nasogastric (NG) tube	9 (32.1%)
Compensatory strategies	12 (42.9%)
Diet texture modification	12 (42.9%)
Feeding posture modification	2 (7.1%)
Rehabilitative exercises	9 (32.1%)
Oromotor exercises	8 (28.6%)
Swallowing manoeuvres	4 (14.3%)
Sensory stimulation	0 (0.0%)

Of 149 patients, forty patients (26.8%) were assessed by speech therapists for their swallowing function due to referral from physicians or self-reported swallowing difficulties. The incidence of dysphagia confirmed by speech therapists through clinical bedside assessment and/or instrumental swallowing assessment in the study cohort was 18.8% (*n* = 28 /149).

##### Dysphagia Symptoms

All dysphagic patients were assessed using clinical bedside assessment by speech therapists. However, 17 out of 28 (60.7%) patients were further evaluated by instrumental assessments including videofluoroscopic swallowing study (VFSS; *n* = 15; 88.2%), flexible endoscopic swallowing study (FEES; *n* = 3; 17.6%) and oesophagogastroduodenoscopy (OGD; *n* = 2; 11.8%). Instrumental assessments revealed that patients had predominantly pharyngeal phase impairments (*n* = 19, 67.9%), including reduced laryngeal excursion and epiglottic retroflexion (*n* = 18, 64.3%), pharyngeal residue (*n* = 14, 50.0%), reduced tongue base retraction (*n* = 12, 42.9%), aspiration (*n* = 11, 39.3%), reduced pharyngeal wall contraction (*n* = 10, 35.7%), impaired upper oesophageal sphincter relaxation (*n* = 10, 35.7%), penetration (*n* = 10, 35.7%) and delayed swallowing reflex (*n* = 7, 25.0%). Importantly, silent aspiration was observed in 6 (21.4%) patients. These patients also exhibited oral phase impairments (*n* = 9, 32.1%), including reduced tongue movement (*n* = 6, 21.4%), abnormal chewing pattern (*n* = 4, 14.3%) and oral residue (*n* = 3, 10.7%), and oesophageal phase impairments (*n* = 5, 17.9%) which included oesophageal dysmotility (*n* = 4, 14.3%) and oesophageal residue (*n* = 2, 7.1%). The dysphagia symptoms are summarised in Table 2.

##### Dysphagia Management

Dysphagia was managed with enteral feeding (*n* = 10, 35.7%), compensatory strategies (*n* = 12, 42.9%) and rehabilitative exercises (*n* = 9, 32.1%). Among patients with enteral feeding, all but one received nasogastric tube feeding. The most common compensatory strategy was diet modification (*n* = 12, 42.9%), while feeding posture modification (chin-tuck) was recommended in 2 patients (7.1%). For rehabilitative exercises, tongue hold manoeuvre (Masako exercise) was the most frequently used oromotor exercise, and Mendelson’s manoeuvre and effortful swallow were used as swallowing manoeuvres. By contrast, no patients received sensory stimulation as part of the dysphagia management.

### Comparisons between Dermatomyositis Patients with and without Dysphagia

Table 3 summarises the characteristics of dermatomyositis patients with and without dysphagia. Compared to dermatomyositis patients without dysphagia, the age of diagnosis was significantly older in patients with dysphagia (*p* = 0.014) with shorter duration of disease (*p* < 0.001). More patients with dysphagia had underlying malignancy (OR [95%CI] = 3.17 [1.34, 7.48], *p* = 0.015) and they had higher mortality rate (OR [95%CI] = 2.90 [1.13, 7.45], *p* = 0.049) than non-dysphagic patients. The duration for CK level to return to normal was significantly shorter in patients with dysphagia (*p* = 0.026). Finally, more patients with dysphagia were treated with IVIg than those without dysphagia (OR [95%CI] = 4.44 [1.36, 14.49], *p* = 0.017).

**Table 3:** Comparisons between dermatomyositis patients with dysphagia and without dysphagia

	With dysphagia (*n* = 28)	Without dysphagia (*n* = 121)	Odds ratio (95%CI)	*p* value
**Demographics**				
Gender (Female)	14 (50%)	84 (69.4%)	0.44 (0.19, 1.02)	0.076
Age of diagnosis (years)	59.5 (30–81)	52 (3–92)	--	*0.014
**Clinical characteristics**				
Disease duration (months)	41 (3–250)	104 (5–394)	--	*<0.001
Number of patients went into ^a^remission	0 (0.0%)	7 (5.8%)	0.96 (0.92, 1.00)	0.584
Number of deaths	9 (32.1%)	17 (14.0%)	2.90 (1.13, 7.45)	*0.049
Underlying malignancy	13 (46.4%)	26 (21.5%)	3.17 (1.34, 7.48)	*0.015
Interstitial lung disease (ILD)	3 (10.7%)	23 (19.0%)	0.51 (0.14, 1.84)	0.411
Bronchiolitis obliterans organizing pneumonia (BOOP)	0 (0.0%)	2 (1.7%)	0.84 (0.04, 17.95)	1.000
Ischaemic heart disease	1 (3.6%)	8 (6.6%)	0.52 (0.06, 4.36)	1.000
Stroke	3 (10.7%)	5 (4.1%)	2.78 (0.62, 12.42)	0.172
**Laboratory characteristics**				
CK level (unit/L) before systemic treatment	426 (44–5775)	295.5 (20–11493)	--	0.119
^b^*Elevated CK level* (n [%])	19 (67.9%)	53 (43.8%)	1.66 (0.66, 4.19)	0.368
*Duration for CK level remission (days)*	31.5 (1–230)	75.5 (1–4261)	--	*0.026
MDA5 positive	3 (10.7%)	14 (11.6%)	0.92 (0.25, 3.44)	1.000
TIF1-γ positive	5 (17.9%)	11 (9.1%)	2.17 (0.69, 6.86)	0.184
ANA level	1:160 (1:80–1:1440)	1:160 (1:40–1:1440)	--	0.665
^+^*Elevated ANA level*	12 (42.9%)	35 (28.9%)	1.84 (0.79, 4.29)	0.178
Anti-dsDNA antibody level (IU/ml)	2.6 (1–7.9)	4.8 (0.6–246.4)	--	0.102
^+^*Elevated anti-dsDNA level*	0 (0.0%)	7 (5.8%)	0.27 (0.01, 4.83)	0.348
**Medications**				
Systemic corticosteroid				
Prednisolone	25 (89.3%)	87 (71.9%)	3.26 (0.92, 11.50)	0.087
Methylprednisolone	4 (14.3%)	5 (4.1%)	3.87 (0.97, 15.47)	0.064
Duration of systemic corticosteroid use (days)	965 (3–6713)	1565.5 (15–8049)	--	0.162
Immunosuppressants				
Azathioprine	5 (17.9%)	36 (29.8%)	0.51 (0.18, 1.46)	0.246
Hydroxychloroquine	13 (46.4%)	60 (49.6%)	0.91 (0.40, 2.08)	0.835
Methotrexate	0 (0.0%)	14 (11.6%)	0.13 (0.01, 2.25)	0.073
Mycophenolate mofetil	1 (3.6%)	8 (6.6%)	0.52 (0.06, 4.36)	1.000
Tacrolimus	0 (0.0%)	2 (1.7%)	0.84 (0.04, 17.96)	1.000
Tocilizumab	0 (0.0%)	1 (0.8%)	1.41 (0.06, 35.51)	1.000
Infliximab	0 (0.0%)	1 (0.8%)	1.41 (0.06, 35.51)	1.000
Rituximab	0 (0.0%)	6 (5.0%)	0.31 (0.02, 5.70)	0.594
Cyclosporine	0 (0.0%)	2 (1.7%)	0.84 (0.04, 17.96)	1.000
Intravenous Immunoglobulin	6 (21.4%)	7 (5.8%)	4.44 (1.36, 14.49)	*0.017

Values are presented in n (%) or median (range) unless otherwise specified. P values were obtained from Mann Whitney *U* or Fisher’s exact tests.*p < 0.05; CK: Creatine Kinase; MDA5: melanoma differentiation-associated protein 5; TIF1-γ: transcription intermediary factor 1-gamma; ANA: anti-nuclear antibody; anti-dsDNA: anti-double stranded DNA; ^a^ Remission is defined as free of systemic treatment; ^b^CK > 355U/L for males and > 161 U/L for females; ANA > 1/80; dsDNA level > 30 IU/ml. These reference values were provided by the local laboratory

### Correlation between Dysphagia and Clinical and Laboratory Characteristics of Dermatomyositis

Dysphagia was positively correlated with the age of diagnosis (*r*[148] = 0.187, *p* = 0.023), mortality (*r*[149] = 0.186, *p* = 0.023), presence of underlying malignancy (*r*[149] = 0.222, *p* = 0.007), use of methylprednisolone (*r*[149] = 0.166, *p* = 0.042) and IVIg (*r*[149] = 0.217, *p* = 0.008) (Table 4). These correlations were considered small. Moreover, there was a small negative correlation between dysphagia and disease duration (*r*[147]=-0.273, *p* < 0.001). There was a trend to significant correlation between dysphagia and gender (*r*[149] = 0.160, *p* = 0.051).

**Table 4:** Correlation between dysphagia and clinical and laboratory characteristics of dermatomyositis

	Pearson’s correlation coefficient, *r*	*p* value
**Demographics**		
Gender	0.16	0.051
Age of diagnosis	0.187	*0.023
**Clinical characteristics**		
Disease duration	-0.273	*<0.001
Number of patients went into ^a^remission	-0.090	0.277
Number of deaths	0.186	*0.023
Underlying malignancy	0.222	*0.007
Interstitial lung disease (ILD)	-0.085	0.301
Bronchiolitis obliterans organizing pneumonia (BOOP)	-0.056	0.497
Ischaemic heart disease	-0.050	0.546
Stroke	0.114	0.166
**Laboratory characteristics**		
CK level before systemic treatment	0.020	0.831
^b^*Elevated CK level*	0.099	0.286
*Duration of CK level remission*	-0.204	0.103
MDA5 positive	-0.011	0.899
TIF1-γ positive	0.111	0.179
ANA level	0.022	0.853
^+^*Elevated ANA level*	0.117	0.155
Anti-dsDNA antibody level	-0.196	0.167
^+^*Elevated anti-dsDNA level*	-0.107	0.195
**Medications**		
Systemic corticosteroid		
Prednisolone	0.157	0.056
Methylprednisolone	0.166	*0.042
*Duration of systemic corticosteroid use*	-0.101	0.318
Immunosuppressants		
Azathioprine	-0.104	0.207
Hydroxychloroquine	-0.025	0.765
Methotrexate	-0.155	0.059
Mycophenolate mofetil	-0.050	0.546
Tacrolimus	-0.056	0.497
Tocilizumab	-0.040	0.632
Infliximab	-0.040	0.632
Rituximab	-0.099	0.232
Cyclosporine	-0.056	0.497
Intravenous Immunoglobulin	0.217	*0.008

CK: Creatine Kinase; MDA5: melanoma differentiation-associated protein 5; TIF1-γ: transcription intermediary factor 1-gamma; ANA: anti-nuclear antibody; anti-dsDNA: anti-double stranded DNA; ^a^ Remission is defined as free of systemic treatment; ^b^CK > 355U/L for males and > 161 U/L for females; ANA > 1/80; dsDNA level > 30 IU/ml. These reference values were provided by the local laboratory; *p < 0.05

### Predictors Dysphagia in Patients with Dermatomyositis

Multivariate logistic regression was performed to ascertain the effects of gender, age of diagnosis, disease duration, presence of underlying malignancy and the use of medications including prednisolone, methylprednisolone, methotrexate and IVIg, on the likelihood of dysphagia in dermatomyositis. The logistic regression model was statistically significant (*χ*^2^ [8] = 37.558, *p* < 0.001). It explained 36.1% (Nagelkerke *R*^2^) of the variance in dysphagia in dermatomyositis and correctly classified 85.1% of cases. Shorter disease duration (OR [95%CI] = 0.99[0.99, 1.00], *p* = 0.045), and the use of prednisolone (OR[95%CI] = 4.43[1.02, 19.27], *p* = 0.047) and IVIg (OR[95%CI] = 6.39[1.14, 35.68], *p* = 0.035) were independent predictors of dysphagia.

## Discussion

This study reviewed the characteristics of dysphagia in patients with dermatomyositis. We found that the incidence of dysphagia was 18.8% in this cohort, with predominant pharyngeal phase impairments. However, only forty (26.8%) of patients reported symptomatic dysphagia during follow-up and were assessed by speech therapists on their swallowing function, suggesting that our observed incidence might be underestimated. Dysphagia was managed mainly by enteral feeding and other compensatory strategies. Our findings showed dysphagia correlated with advanced age of diagnosis, mortality, presence of underlying malignancy and the use of methylprednisolone and IVIg, and shorter disease duration. Finally, shorter disease duration and the use of prednisolone and IVIg were independent predictors of dysphagia. Taken together, our findings highlighted the presence of dysphagia in dermatomyositis can be significant, especially 21% had silent aspiration which may result in more serious sequelae such as aspiration pneumonia. We have identified several risk factors for dysphagia among patients with dermatomyositis, which merit further discussions.

### Prevalence of Dysphagia and Swallowing Assessments

Our results showed that the incidence of dysphagia among patients with dermatomyositis was 18.8%, which is comparable to the prevalence reported in Asian countries (China: 11.7–33.7% [17–22]; Japan: 14.1–28.0% [23–25]; Singapore: 14.3% [26]; South Korea: 20.4–32.7% [27–29]), but lower than that reported in European countries (14.4–66.7% [30–34]) and the United States (8.9–84.2% [35–37]). A possible explanation for the lower prevalence in Asian countries is the differences in diets (more likely to be smaller in amount and size of foods) and genetic makeups such that genes associated with dysphagia in dermatomyositis may be less prevalent in Asian populations than Caucasian populations. However, such association have not been reported in the literature to date.

The incidence of dysphagia may be higher than reported because only approximately one-quarter (26.8%) of patients were assessed by speech therapists, and even less (11.4%) were assessed using instrumental swallowing assessments. Physicians rely on patients’ self-report of swallowing difficulties at the time of dermatomyositis diagnosis and refer to speech therapists for swallowing assessments only when patients complain of swallowing difficulties. However, patients tend to underestimate the severity of dysphagia. The study by Cox et al., found that dysphagia was prevalent (65%) among patients with inclusion body myositis (IBM), but only less than half of the patients complained about their swallowing problems with their physicians [38]. Another study also found that patients with IIM do not report their swallowing problems unless specifically asked by their physicians [39]. These findings suggested that patients with IIM may underreport their swallowing difficulties, which supported our speculations that the observed dysphagia prevalence may be underestimated.

Instrumental swallowing assessment is considered as the “gold standard” for dysphagia diagnosis because clinical bedside assessment may overlook covert signs of dysphagia such as silent aspiration, leading to inaccurate diagnosis. In our study, among patients who were assessed by speech therapists, only 60.7% were assessed using instrumental assessments. An early study have reported discrepancy in detecting dysphagia symptoms between clinical bedside and instrumental assessments [40]. Therefore, relying solely on either assessment may under- or over-estimate the severity of dysphagia. Our findings showed that patients predominately exhibited pharyngeal phase impairments while some (17.9%) exhibited oesophageal impairments. However, previous studies suggested that oesophageal impairments were frequent in patient with dermatomyositis or polymyositis (84%) [41–43]. This discrepancy can be attributed to the difference in the type of instrumental swallowing assessments used to assess the oesophageal phase of swallowing in our study cohort. The oesophageal motility of most patients was assessed using VFSS with an oesophageal evaluation, while that of 2 patients were further assessed with OGD. OGD allows visualisation of contractions of the oesophagus, which may be used as an additional instrumental assessment that provides supplementary information regarding the oesophageal motility. An early study by Jacob et al., found that some patients who showed decreased motility in distal or proximal oesophagus during manometric evaluation did not show abnormalities in the same regions on VFSS [41]. Therefore, it is possible that the prevalence of oesophageal impairments may differ based on the type of instrument used.

Taken together, our findings reflected that there may be lack of awareness of dysphagia among patients and physicians. More proactive enquiry of dysphagia and formal swallowing assessments during initial medical consultation among patients with dermatomyositis should be considered especially for patients with advanced age, and underlying malignancy. The timing for screening is also important, especially at initial onset of DM (short disease duration of DM) and for patients with more severe disease (as reflected by more frequent use of high dose systemic corticosteroid and IVIg where myositis symptoms were more prominent).Therefore, it is highly recommended that the assessment of swallowing function should be included in the normal assessment battery given to these patients. With respect to screening for dysphagia, patient-reported questionnaires such as Sydney Swallowing Questionnaire (SSQ) [44, 45] should be used to allow systematic and detailed screening of swallowing difficulties. Mulcahy et al., demonstrated that patient-reported questionnaires correlated with findings from instrumental swallowing assessments in patients with IIM, suggesting the value of these questionnaires in identifying early symptoms of dysphagia [39]. Moreover, swallowing function of patients with dermatomyositis should be monitored throughout the course of the disease using both clinical bedside and instrumental swallowing assessments.

### Dysphagia Management

The most common strategy for dysphagia management in our study cohort was compensatory strategies, including enteral feeding. Compensatory strategies aim to ensure swallowing safety by modification of feeding environment and do not involve active participation of patients. These strategies therefore do not improve swallowing function, instead they aim to maximize swallowing safety within the restrictions of the patient’s ability. It is unclear why rehabilitative strategies such as oromotor exercises were not used more often in our study cohort. It is possible that given dysphagia typically resolves with the resolution of the disease, speech therapists may employ a passive approach to maintain the patient’s swallowing safety while waiting for disease resolution. However, this approach can lead to delayed management which may be detrimental to the patient’s quality of life, particularly those with prolonged enteral feeding [46]. Patients with enteral feeding may experience poor quality of life due to negative body image, gustatory deprivation and the loss of social contacts [46]. Moreover, a systematic review reported that enteral feeding is not recommended in patients other than stroke, chronic liver disease, perioperative, critically ill or low birth weight patients [47]. Therefore, we recommend that dysphagia in dermatomyositis should be managed with a proactive approach by adopting rehabilitative exercises to promote functional gains in swallowing, in parallel to management of dermatomyositis.

### Underlying Malignancy, Mortality and Dysphagia

Concurrent with previous studies, we found that dysphagia is correlated with underlying malignancy and mortality [48]. Previous studies have suggested that dysphagia is associated with poorer prognosis with worse general condition of the disease [49, 50]. Moreover, Airio et al., found that patients with dermatomyositis had higher risk of dying from underlying malignancy [51]. Therefore, it is not surprising that dysphagia is found to be associated with increased mortality. Nonetheless, our finding highlighted the association between dysphagia and underlying malignancy, such that screening for these conditions should be performed early and regularly for timely detection and intervention.

### Old age, Shorter Disease Duration and Dysphagia

We found that shorter disease duration was a predictor of dysphagia. In our study cohort, none of the patients with dysphagia went into remission. Therefore, the shorter disease duration was likely due to older age at diagnosis and higher mortality rate. It may also reflect greater disease severity at the time of presentation or more rapid progression of the disease. Furthermore, sarcopenia, which is a syndrome of progressive loss of skeletal muscle mass and strength, is common among elderly and is associated with increased risk of dysphagia [52]. Older patients may exhibit presbyphagia before dermatomyositis such that they have less flexible compensatory mechanisms for swallowing. When the swallowing muscles are affected by dermatomyositis-related inflammation, they may be less likely to compensate for the deficits and more likely to exhibit symptoms compared to younger patients.

### Use of Prednisolone and IVIg as Independent Predictor for Dysphagia

We found that the use of prednisolone and IVIg were independent predictors of dysphagia. Systemic corticosteroid is typically used as the first line treatment for dermatomyositis [53]. However, some patients may not be responsive to corticosteroid therapy such that immunosuppressants or IVIg may be added [53]. Studies have found that dermatomyositis or polymyositis patients with oesophageal dysphagia who were resistant to prednisolone showed improvements in swallowing function following IVIg treatments [43, 49]. It is possible that the use of both prednisolone and IVIg may reflect greater severity of dermatomyositis than those who received corticosteroid treatment only. These patients may have increased muscle weakness and hence increased risk of dysphagia.

### Limitations of the Study

Our study had several limitations. First, as it was a retrospective study, patients who did not complain of dysphagia was not screened routinely by speech therapists. Moreover, there is a lack of systematic assessment of swallowing functions using instrumental assessment techniques. This may result in underestimation of the prevalence of dysphagia in our cohort. Second, the findings from this single centre study may not be representative of the entire Hong Kong population. Moreover, the sample size for multivariate logistic regression analysis was small (*n* = 28) and multiple comparisons were not accounted for such that the findings may be overestimated. Finally, since the study reviewed medical records spanning a period of 10 years, the clinical practice including documentation of consultation notes, diagnostic criteria and the use of medications may have changed throughout this period. This may result in inconsistent reports across patients.

## Conclusions

Our study retrospectively reviewed the characteristics of dysphagia among patients with dermatomyositis in Hong Kong. We found that the incidence of dysphagia was 18.8%, although such data may be an underestimation given the lack of routine swallowing assessments in our study cohort. We found that dysphagia is associated with presence of underlying malignancy, increase mortality rate, shorter duration of disease and older age at diagnosis. Moreover, shorter disease duration and the use of prednisolone and IVIg were independent predictors of dysphagia in dermatomyositis. Taken together, our findings call for routine dysphagia screening as part of the assessment battery for dermatomyositis in clinical practice.

## Data Availability

The data that support the findings of this study are available from the corresponding author upon reasonable request.

## Abbreviations

ANA: Anti-nuclear antibody
Anti-dsDNA: anti-double stranded DNA
BOOP: Bronchiolitis obliterans organizing pneumonia
CDARS: Clinical data analysis and recording system
CI: Confidence interval
CK: Creatine kinase
FEES: Flexible endoscopic swallowing study
ICD-9-CM: International Classification of Diseases, Ninth Revision, Clinical Modification
IIM: Idiopathic inflammatory myopathy
ILD: Interstitial lung disease
IVIg: Intravenous immunoglobulin
MDA5: Melanoma differentiation-associated protein 5
OGD: Oesophagogastroduodenoscopy
OR: Odds ratio
SD: Standard deviation
SSQ: Sydney Swallowing Questionnaire
ST: Speech therapists
TIF1-γ: Transcription intermediary factor 1-gamma
VFSS: Videofluoroscopic swallowing study
